# Resveratrol serves as a protein-substrate interaction stabilizer in human SIRT1 activation

**DOI:** 10.1038/srep38186

**Published:** 2016-11-30

**Authors:** Xuben Hou, David Rooklin, Hao Fang, Yingkai Zhang

**Affiliations:** 1 Department of Medicinal Chemistry and Key Laboratory of Chemical Biology of Natural Products (MOE), School of Pharmacy, Shandong University, Jinan, Shandong, 250012, China; 2 Department of Chemistry, New York University, New York, New York, 10003, United States; 3 NYU-ECNU Center for Computational Chemistry at NYU Shanghai, Shanghai, 200062, China

## Abstract

Resveratrol is a natural compound found in red wine that has been suggested to exert its potential health benefit through the activation of SIRT1, a crucial member of the mammalian NAD^+^-dependent deacetylases. SIRT1 has emerged as an attractive therapeutic target for many aging related diseases, however, how its activity can only be activated toward some specific substrates by resveratrol has been poorly understood. Herein, by employing extensive molecular dynamics simulations as well as fragment-centric topographical mapping of binding interfaces, we have clarified current controversies in the literature and elucidated that resveratrol plays an important activation role by stabilizing SIRT1/peptide interactions in a substrate-specific manner. This new mechanism highlights the importance of the N-terminal domain in substrate recognition, explains the activity restoration role of resveratrol toward some “loose-binding” substrates of SIRT1, and has significant implications for the rational design of new substrate-specific SIRT1 modulators.

The Sir2 (silent information regulator 2) protein family, also called sirtuins, are conserved NAD-dependent protein deacetylases in a range of organisms and play important roles in many biological processes^1^,^2^,^3^,^4^. The yeast Sir2 has been found to regulate various metabolic pathways and mediate many beneficial effects of calorie restriction (CR), such as lifespan extension^5^,^6^. Among seven mammalian sirtuins (SIRT1-7), the best-characterized SIRT1 is the closest homologue to yeast Sir2. Different from yeast Sir2 that primarily deacetylates histone H4, SIRT1 deacetylates a variety of substrates including histones, p53, forkhead box protein (FOXO), nuclear factor (NF)-κB, and peroxisome proliferator-activated receptor gamma coactivator 1α (PGC-1α)^7^. In line with this, SIRT1 plays important roles in many aging-related biological processes such as growth regulation, energy metabolism, stress response, and endocrine signaling^7^. Evidence derived from *in vivo* studies further confirmed that SIRT1 mediates effects of CR in mice, including lifespan extension^5^,^8^,^9^,^10^,^11^,^12^,^13^. In addition, SIRT1 protein levels are increased in response to CR, and genetic ablation of SIRT1 prevents many health benefits of a CR diet^14^,^15^,^16^,^17^,^18^. Accordingly, there has been significant interest in developing SIRT1 activators as calorie restriction mimetics for the treatment of aging-related diseases^4^.

Resveratrol, a plant polyphenol, was initially identified as a small molecule SIRT1 activator through *in vitro* screening and many studies have linked the health benefits of resveratrol to modulation of SIRT1 activity^19^,^20^,^21^,^22^,^23^. For example, resveratrol has been shown to extend the life span of yeast^24^, flies^25^ and mice^26^ by mimicking the beneficial effects of CR in a Sir2-dependent manner^26^. Moreover, resveratrol produces pharmacological changes consistent with SIRT1 overexpression in cellular tests^4^,^26^. It was initially demonstrated that resveratrol could significantly increase binding affinity of substrate by lowering its Michaelis constant (*K*_*m*_) value without affecting the overall turnover rate (*V*_*max*_) of SIRT1^24^,^27^,^28^. However, subsequent experiments found that the observed activation effect is dependent on the use of the fluorescent substrate (p53AMC) in the enzyme activity assay, and resveratrol did not enhance SIRT1 activity against some well-known native SIRT1 substrates (e.g. p53)^27^,^28^,^29^,^30^. This raised significant controversy regarding whether or not resveratrol is truly a direct SIRT1 activator. Recent biological studies have revealed that resveratrol does activate SIRT1 toward certain substrates containing a bulky hydrophobic group, such as a 7-amino-4-methylcoumarin (AMC) moiety or a tryptophan residue, directly adjacent to the acetylated Lys at the +1 position. And a recent crystal structure of SIRT1 in complex with p53AMC revealed the co-binding of three resveratrol molecules in the N-terminal domain (NTD) of SIRT1, mediating the protein-substrate interaction^31^. In addition to this artificial p53AMC substrate, resveratrol is also known to increase SIRT1 activity toward some specific non-fluorophore substrates, such as p53W and SF38A^32^,^33^. Up to now, the most widely accepted mechanism regarding SIRT1 activation is the allosteric mechanism^31^,^34^: the binding of resveratrol leads to a conformational change in the NTD of SIRT1 that promotes tighter binding between SIRT1 and the substrate, as shown in Fig. 1a. Since the NTD is unique to yeast Sir2 and mammalian SIRT1, this may explain the reason why resveratrol is an isoform-selective sirtuin activator (Fig. 1b).

However, many molecular details regarding this allosteric mechanism remain unclear, and there are limitations to the current substrate-dependent allosteric mechanism of SIRT1 activation. For example, it is unable to explain why resveratrol confers no activation toward the well-known native p53 substrate, thus the substrate specificity underlying SIRT1 activation has remained elusive. Moreover, whether SIRT1 activation is actually dependent on a resveratrol induced protein conformational change is not itself clear. Although the activated SIRT1 conformation has been reported in the crystallographic study by Cao *et al*.^31^, the inactivated NTD-containing SIRT1 structure without resveratrol binding is still uncharacterized. In another SIRT1 crystal structure, recently determined by Dai *et al*., the NTD was observed to be distant from the substrate despite being bound to a synthetic SIRT1 activator^32^.

In this work, in order to elucidate how SIRT1 can be activated by resveratrol, we performed a total of 7-μs explicit-water molecular dynamics (MD) simulations as well as fragment-centric topographical mapping analysis^35^ using different SIRT1-substrate systems, with and without resveratrol bound. To make our computational study comparable to corresponding experimental observations, three different p53-derived substrates (p53, p53W and p53AMC) were used in our simulations (Table 1). Based on computational results, we propose a new substrate-dependent SIRT1 activation mechanism, in which resveratrol serves as a protein-substrate interaction stabilizer rather than an allosteric modulator. Different from the classical allosteric mechanism that suggests resveratrol-induced protein conformational change as the origin of SIRT1 activation^2^,^4^,^28^,^29^,^34^,^36^, we found that the tight substrate binding of SIRT1 is dependent on proper NTD-substrate interactions. It is likely that reveratrol may only restore tight binding between SIRT1 and some specific “loose-binding” substrates, such as mutated substrates due to aging. Taken together, the newly proposed protein-substrate interaction stabilization mechanism further improves our understanding of substrate-dependent SIRT1 activation and provide an invaluable model for design of novel substrate-dependent SIRT1 activators.

## Results

### Native p53 substrate binds tightly with SIRT1 in the absence of resveratrol

According to experimental data^27^,^28^,^29^,^31^,^34^, resveratrol can activate SIRT1 toward either p53AMC or p53W, but has no effect toward the native p53 acetyl peptide. Here, by carrying out 5 independent 0.2-μs simulations for each of 7 SIRT1 systems (a total of 7-μs all-atom MD simulations) as well as binding interface analysis, we studied SIRT1-substrate recognition as well as the role of resveratrol in substrate binding related to SIRT1 activation. The details for all simulations in the current study are listed in Supplementary Table S1.

**Figure 1:**
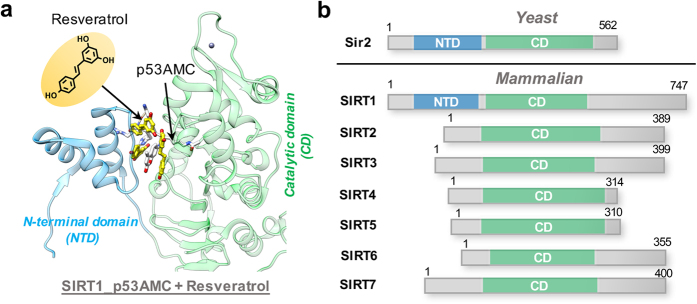
The specific NTD domain is essential for the substrate-dependent SIRT1 activation by resveratrol. (**a**) Crystal structure (PDB: 5BTR^31^) of SIRT1 with p53AMC substrate (white) and three resveratrol (yellow). SIRT1 is presented in ribbon with NTD colored in cyan and CD colored in green. (**b**) Comparison of yeast Sir2 and seven mammalian sirtuins (SIRT1-7). The conserved, catalytic domain (CD) that all sirtuins have in common is colored in green. The N-terminal domains (NTDs) that are unique to yeast Sir2 and mammalian SIRT1 are colored in cyan. Numbers refer to amino acid residues in the proteins.

**Figure 2:**
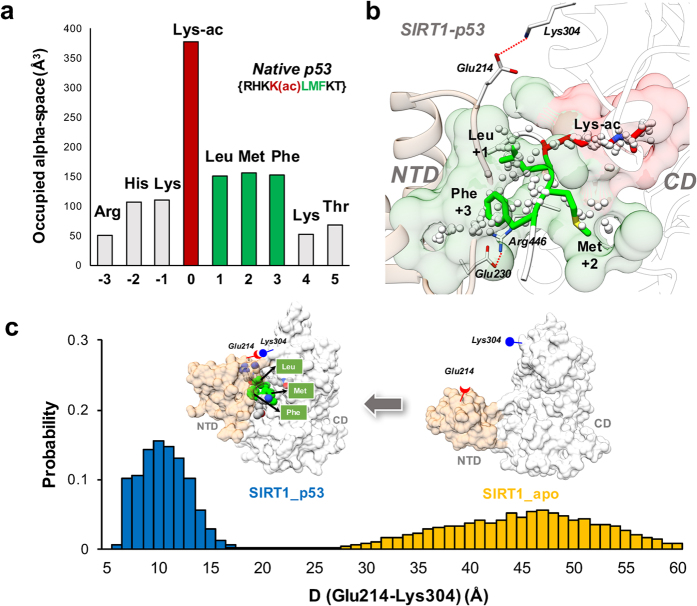
The native p53 substrate binds tightly with SIRT1 in the absence of resveratrol. (**a**) Sequence of native p53 substrate and the average occupied alpha-space on SIRT1, by residue. The data bar of Lys-ac is highlighted in red and that of Leu (+1), Met (+2) and Phe (+3) are highlighted in green. (**b**) Binding pocket analysis of native p53 substrate using the representative conformation from MD simulations. The Lys-ac and its corresponding pocket is colored in red. Leu (+1), Met (+2) and Phe (+3) and their corresponding pockets are colored in green. Alpha-atoms, which represent the pocket space in SIRT1, are depicted at small white spheres. SIRT1 is presented in cartoon with NTD colored in tan and CD colored in transparent white. (**c**) Comparison of NTD-CD distances of apo SIRT1 (yellow) and SIRT1_p53 complex (blue). The distance between NTD and CD was measured using the mass center of two residues (Glu214 and Lys305) in the top of each domain. The representative conformation for each system is shown in surface with NTD colored in tan and CD colored in white. The native p53 substrate is shown in sphere with three residues Leu, Met and Phe highlighted in green.

Heretofore, how SIRT1 recognizes the native p53 acetyl peptide substrate has been elusive. Using the crystal structure of SIRT1 in complex with p53AMC substrate and resveratrol (PDB: 5BTR^31^) as a template, we constructed and investigated the binding complex of the native p53 acetyl peptide substrate to SIRT1 using a total of 1-μs MD simulations. As shown in Fig. 2, SIRT1 does bind the native p53 substrate tightly in the absence of resveratrol through complementary pockets in both the NTD and CD. We analyze the native p53 binding interface of SIRT1 using *AlphaSpace*^35^, whose key attractive feature is its capability to detect the fragment-centric modularity at the protein surface and to characterize the large protein binding interface as a set of localized, fragment-targetable interaction pockets. Interestingly, the acetyl lysine (Lys-ac) registers the highest pocket occupation for p53 residues, followed by three C-terminal residues (Leu, Met, and Phe) (Fig. 2a). Consistent with the pocket analysis results, the calculated interaction energies of p53 with SIRT1 using MM/GBSA method further highlighted the importance of Lys-ac, Leu, Met, and Phe in substrate binding (Supplementary Fig. S1). As shown in Fig. 2b, the Lys-ac binds into a narrow, tube-like pocket near the SIRT1 catalytic site, which is the common binding mode of Lys-ac in sirtuin family proteins^37^. Because the C-terminal residues define the variation between the three p53-derived substrates, we focus on the binding pockets of Leu (+1), Met (+2) and Phe (+3) in p53. The binding pocket for Met (+2), in the catalytic domain, adjacent to the Lys-ac binding pocket, is also observed in other sirtuin-p53 crystal structures (PDB: 1MA3^38^ 2H2F^37^ and 4ZZJ^32^). The substrate pockets in the NTD are most important for specific SIRT1-substrate recognition, as this special NTD is found in SIRT1 alone among seven human sirtuins (Fig. 1b). In comparison with the +3 position Phe residue that binds to a large pocket in the NTD, the +1 position Leu binds to a smaller NTD pocket and also interacts with some residues in the CD (Fig. 2b). Besides the previously reported Glu230-Arg446 salt-bridge interaction^32^,^34^, our MD simulations reveal another salt-bridge interaction between the NTD and CD (Glu214-Lys304). Although the probability of the solvent-exposed Glu214-Lys304 salt-bridge formation (0.13) is lower than that of the more shielded Glu230-Arg446 salt-bridge (0.44), the proximity of Glu214 and Lys304 (around 10 Å) is observed when p53 bound NTD and CD tightly (Fig. 2c).

We further performed MD simulations of apo SIRT1 using the same crystal structure to set up the initial simulation system. As shown in Fig. 2c, the NTD is distant from the CD in the apo state of SIRT1 and displays high flexibility (Supplementary Fig. S2). In this open state, the acetyl substrate can easily get access to the catalytic site directly, as the Lys-ac pocket is widely open to accommodate the entrance of substrate (Supplementary Fig. S3). On the other hand, the hydrophobic pockets in the NTD can still be detected in the apo state but are less well organized than in the SIRT1-p53 binding complex, indicating that the binding of substrate may induce the formation of the complementary pockets in the NTD (Supplementary Fig. S3). Interestingly, the Glu230-Arg446 salt-bridge interaction is still detected in the apo-SIRT1 system with a similar probability value (0.45) as that of the SIRT1/p53 system (0.44). However, there is no observable Glu214-Lys304 salt-bridge interaction in the apo state. Accordingly, this distance, between residue Glu214 and Lys304, was selected as a metric to represent the closeness between the NTD and CD in the current study. When Glu214 is in proximity to Lys304, we observe the substrate to be tightly bound with SIRT1 through interactions with both NTD and CD (Fig. 2c).

In the previously proposed allosteric SIRT1 activation mechanism by resveratrol, a key component is that the binding of resveratrol leads to a conformational change of SIRT1, such as the closure of NTD (Fig. 1a). Here our findings indicate that the closure of NTD is not dependent on resveratrol. As described above, the native p53 substrate can readily stabilize the closure of NTD with a tight NTD-substrate interaction (Fig. 2 and Videos S1-S2) without the presence of resveratrol. These results not only elucidate the structural basis for SIRT1 recognition of the native p53 substrate from a binding pocket perspective, but also set the stage to further investigate the SIRT1 activation mechanism by resveratrol.

### Resveratrol restores tight NTD-p53AMC/p53W interaction by creating a new complementary pocket for bulky groups in the +1 position

**Figure 3:**
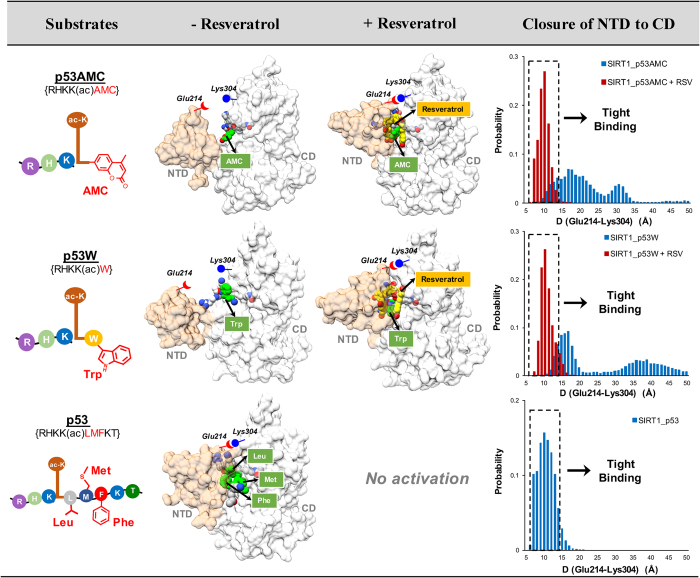
Resveratrol stabilized the interactions between substrate p53AMC/p53W and NTD domain of SIRT1. Three p53-derived substrates are shown as cartoon with differences in chemical structures colored in red. Representative structures from MD simulations using three different substrates with and without resveratrol. SIRT1 protein is shown in surface with NTD colored in tan and CD colored in white. Substrates are presented in grey sphere, with the variable motifs colored green. Resveratrol molecules are presented using yellow sphere. Distances between Glu214 (NTD) and Lys304 (CD) are used to represent the closeness between NTD and CD. The tight binding is achieved when NTD closes on substrate and CD.

For the simulated SIRT1/p53 complex without resveratrol, the tight binding of substrate to SIRT1 is achieved by interacting with both NTD and CD (Fig. 2 and Videos S1-S2). However, this is not the case for either p53AMC or p53W peptide. As shown in Fig. 3, our MD simulations demonstrate that neither p53AMC nor p53W can form stable interactions with the NTD of SIRT1 in the absence of resveratrol, with the distance between NTD (Glu214) and CD (Lys304) fluctuating from 10 Å to 50 Å (Fig. 3 and Videos S3-S6). Remarkably, the binding of resveratrol dramatically stabilizes the interaction between the NTD and p53AMC/p53W, with the NTD-CD distance observed to fluctuate around 10 Å (tight binding state, as in the binding complex of SIRT1-p53 without resveratrol). By employing MM/GBSA methodology (see Supplementary for details), we further calculated the interaction energies for the three p53-derived substrates with and without resveratrol bound. The interaction energies of p53AMC and p53W were significantly increased with resveratrol bound, whereas no apparent changes were observed for resveratrol with native p53 (Supplementary Fig. S4). Thus our simulations are consistent with experimental results^27^,^28^,^29^,^31^,^34^: the native p53 substrate alone binds tightly with SIRT1, however, either p53AMC or p53W needs resveratrol to restore tight binding to SIRT1 (Supplementary Figs S5–S7).

**Figure 4:**
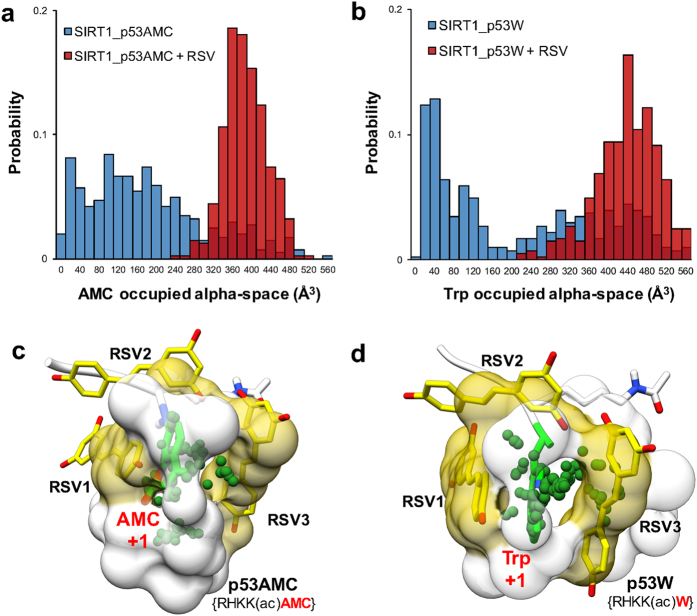
Pocket analyzes of +1 residues in p53AMC and p53W during MD simulations. Occupied alpha-space by AMC and Trp during simulation using p53AMC (**a**) and p53W (**b**) with and without resveratrol. Binding pocket for AMC and Trp (green) in p53AMC (**c**) and p53W (**d**) with resveratrol (yellow) bound.

Our next objective is to elucidate *how* resveratrol restores tight NTD-p53AMC/p53W interaction. Similar resveratrol binding modes were observed for the p53AMC system and the p53W system, including three resveratrol molecules (RSV1, RSV2 and RSV3) binding into the gap between SIRT1 and the substrate (Supplementary Fig. S8). The three resveratrol molecules interact directly with the large hydrophobic groups in the +1 position of the substrate (AMC group of p53AMC and Trp of p53W). Compared with the high flexibilities of AMC and Trp during MD simulation without activator, binding of resveratrol significantly limits the motion of AMC and Trp (Supplementary Fig. S9). We analyzed the binding pocket of the AMC and Trp groups throughout the MD simulations by fragment-centric topographical mapping using *AlphaSpace*^35^. Resveratrol significantly increases the occupied pocket alpha-space for both AMC and Trp, indicating tight binding of these groups by well-defined pockets (Fig. 4a and b). Importantly, three resveratrol molecules directly contribute to the formation of new binding pockets for AMC and Trp, which restore the tight interaction between p53AMC/p53W and SIRT1 (Fig. 4c and d). On the other hand, neither AMC nor Trp groups can find complementary binding pockets in the NTD during MD simulation in the absence of resveratrol, resulting in an improper NTD-p53AMC/p53W interaction, underscoring these as “loose-binding” substrates of SIRT1 (Supplementary Fig. S10). Thus, the formation of new binding pockets for the AMC and Trp groups is a critical factor in SIRT1 activation by resveratrol. Resveratrol can restore tight NTD-substrate interaction by forming a complementary pocket for the large hydrophobic residues (AMC and Trp) in the +1 position of p53AMC and p53W, as shown in Fig. 4c and d.

**Figure 5:**
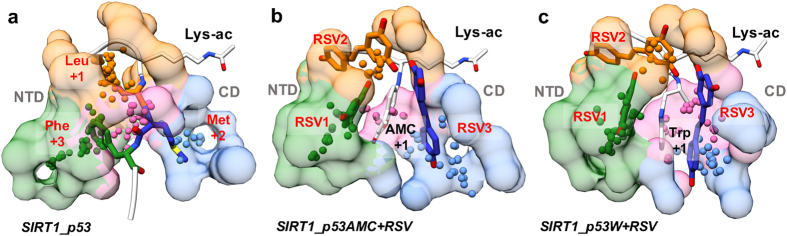
Comparison of the binding pockets of p53 (**a**), p53AMC/RSV complex (**b**) and p53W/RSV complex (**c**). The Leu, Met, and Phe residues in p53 substrate and three resveratrol molecules in p53AMC/RSV complex and p53W/RSV complex are presented in sticks and colored in orange, blue and green, respectively. Residues lys-ac, AMC and Trp are shown as white thin sticks. Other residues in three substrates are shown as white ribbon. Side-chain pockets for three residues (Leu, Met, and Phe) in p53 system and pockets for three resveratrol molecules (RSV1, RSV2, and RSV3) in p53AMC/RSV complex and p53W/RSV complex are presented as alpha-atom spheres and transparent surfaces, using the same color as each residue (or as the resveratrol molecules). Backbone pocket for three p53 residues and pockets for +1 residues (AMC and Trp) in p53AMC and p53W are presented as pink alpha-atom spheres with transparent surfaces.

As mentioned before, we observed that native p53 substrate can form stable interaction with NTD in the absence of resveratrol binding (Fig. 2). Interestingly, the natural pockets of SIRT1 to accommodate Leu (+1), Met (+2) and Phe (+3) residues of the native p53 substrate are similar to the binding pockets for RSV2, RSV3, and RSV1, respectively (Fig. 5). Thus not surprisingly, in our MD simulations of SIRT1-p53 complex in the presence of resveratrol, all three RSV molecules are observed to run out of their initial pockets and do not form stable interactions with the p53 substrate (Supplementary Figs S5, S10 and S11). These results indicate that resveratrol molecules and the native p53 peptide are competitive toward similar binding pockets, which would explain why resveratrol confers no activation effect toward native p53.

### Protein-substrate interaction stabilization-based SIRT1 activation mechanism

**Figure 6:**
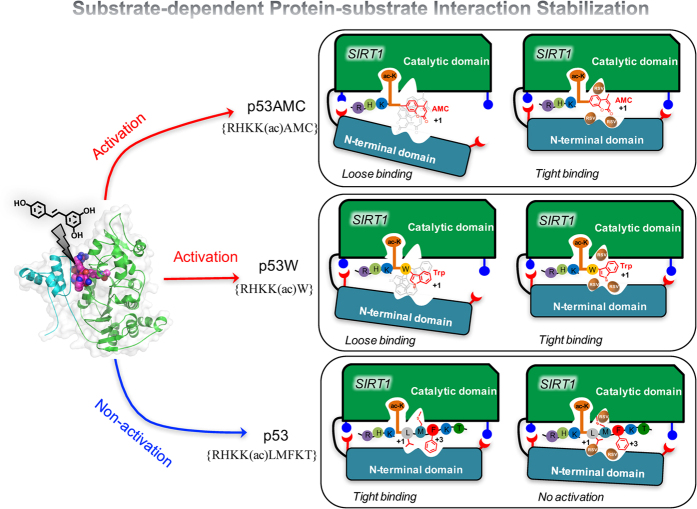
Proposed substrate-dependent activation mechanism of SIRT1 by resveratrol.

Taking all results above together, here we propose a detailed substrate-dependent SIRT1 activation mechanism by resveratrol. This detailed mechanism not only explains why resveratrol fails to activate the native p53 substrate, but also elucidates how resveratrol promotes NTD-p53AMC/p53W interaction. As summarized in Fig. 6, SIRT1 already contains natural binding pockets for native p53 substrate, and binds native p53 substrate tightly in the absence of resveratrol through these complementary pockets in both the NTD and CD. Meanwhile, resveratrol molecules are competitive binders toward very similar pockets. Thus resveratrol cannot further enhance the binding between SIRT1 and the native p53 substrate. On the other hand, neither p53AMC nor p53W can form stable interaction with NTD due to the large hydrophobic groups in the +1 position (AMC and Trp). Importantly, the binding of resveratrol into the native p53 pocket space creates a new, larger binding pocket that can engage the bulky hydrophobic groups at the +1 position to restore the tight NTD-substrate interaction. We can see that this protein-substrate interaction stabilization mechanism highlights the importance of the NTD in SIRT1-substrate recognition, and would suggest that resveratrol may only activate “loose-binding” SIRT1 substrates (e.g. p53AMC, p53W) by restoring the NTD-substrate interactions.

## Discussion

As the first identified SIRT1 activator, resveratrol has gained interest due to its therapeutic benefit for various diseases, especially aging-related disease. Although recent studies have presented that resveratrol can directly activate SIRT1 towards specific substrates through an allosteric mechanism^34^,^39^, several experimental observations could not be explained.

In the current study, based on multiple extensive MD simulations as well as fragment-centric topographical mapping, we have proposed a detailed mechanism for substrate-dependent SIRT1 activation: resveratrol mainly acts as a protein-substrate stabilizer and can enhance NTD-substrate interaction for “loose-binding” substrates (e.g. p53AMC, p53W). The “loose-binding” of p53AMC and p53W comes from poor pocket complementarity at the surface and thus high flexibility of AMC and Trp upon binding SIRT1. Resveratrol can directly interact with p53AMC and p53W and enhance their binding with SIRT1 by forming a new binding pocket for AMC and Trp. To the best of our knowledge, our MD simulation with topographical mapping is the first effort to describe the complementary interaction between native p53 and NTD without the presence of resveratrol and to address why resveratrol cannot further strengthen the binding between SIRT1 and the native p53 acetyl peptide. Importantly, we found the binding pockets for three resveratrol are similar to those utilized by three residues in the native p53 substrate. Our newly proposed protein-substrate interaction stabilization-based mechanism of SIRT1 activation is consistent with current experimental results and fills the gap in the previously proposed allosteric mechanism.

Although resveratrol has been reported to possess biological effects related to SIRT1 modulation at the cellular level, the substrates involved in these processes are still unclear. Our study suggests that SIRT1 activators mainly enhance the binding of “loose-binding” substrates, especially those possessing improper interactions with NTD. Therefore, the *in vivo* effects of resveratrol may come from the activation towards some less active SIRT1 substrates, such as some mutated substrates due to aging.

It is not surprising the NTD plays an important role in SIRT1 activation mechanism. Endogenous SIRT1 activator Lamin A and AROS have been reported to target the NTD and enhance the deacetylation activity of SIRT1 towards p53^40^,^41^. The binding of Sir4 to NTD of Sir2 also modulates the deacetylation activity^42^. Similar to resveratrol, these endogenous proteins may also stimulate SIRT1 activity through stabilization of protein-substrate interactions.

Targeting protein-protein interactions (PPIs) has emerged as a promising therapeutic strategy^43^,^44^. Different from PPI inhibition, stabilization of PPIs is an underexplored concept in drug discovery^45^. For example, rapamycin has been reported to inhibit mTOR, another key modulator of ageing and age-related disease, by stabilizing the PPI between mTOR and FKBP12^46^,^47^,^48^,^49^. Here our computational results demonstrate that resveratrol molecules stabilize the interactions between SIRT1 and specific substrate peptides by forming a new binding pocket. This finding has significant implications for the rational design of new substrate-specific SIRT1 stabilizers.

## Method

### Structure preparation

For each of 9 SIRT1 simulation systems, the initial SIRT1 structure was the same as in the crystal structure of 5BTR, the first structure of SIRT1 with fluorophore-tagged peptide p53AMC and resveratrol^31^. Although the crystal structure of full-length SIRT1 is still unknown, the engineered SIRT1 in 5BTR is biochemically equivalent to the full-length enzyme with respect to basal catalytic activity and activation by activators^31^,^32^. The protonation states of charged residues were determined at constant pH 7 based on pKa calculations via the PDB2PQR server^50^. Specially, the conformation and protonation state of catalytic residue His363 is determined based on our previous QM/MM study^51^. The NAD^+^ conformation was derived from PDB:4ZZJ^33^ and modification was made to convert the carbon atom to oxygen. The initial structure of the native p53 substrate was derived form PDB:1MA3 (crystal structure of Sir2-p53 complex)^38^. Initial structure of the p53W peptide was modeled by mutating the AMC in p53AMC to Trp using the *tleap* module in AmberTools 15.

### Molecular dynamics simulations

All MD simulations were preformed with Amber14 molecular dynamics package^52^, employing the Amber14SB force field^53^ for the protein and the TIP3P model for water molecules. Parameters for zinc were derived from the Zinc AMBER Force Field (ZAFF) developed by Merz group^54^. Force field parameters for acetyl lysine and NAD^+^ are obtained from the literature^55^,^56^. Partial charges for the AMC moiety and for resveratrol were fit with HF/6-31 G(d) calculations using Gaussian 09 package^57^ without geometry optimization. The RESP module in the Amber package was employed to fit the charges to each atomic center^58^,^59^. Each system was neutralized with Na^+^ counterions and solvated with explicit TIP3P water in a rectangular periodic box with 12.0 Å buffer using the *tleap* module within AmberTools 15. The solvated systems consisted of ∼65 000 atoms. The Particle-Mesh Ewald method with 12.0 Å cutoff for the non-bonded interactions was used in the energy minimizations and MD simulations. After a series of minimizations and equilibrations (see Supplementary), standard molecular dynamics simulations were performed on GPUs using the CUDA version of PMEMD (Particle Mesh Ewald Molecular Dynamics)^60^,^61^. Each simulation was carried out for 200-ns with periodic boundary condition and snapshots are saved every 10 ps for analysis. The SHAKE algorithm was applied to constrain all bonds involving hydrogen atoms. A time step of 2 fs was used, and the Berendsen thermostat method has been used to control the system temperature at 300 K. All other parameters were default values.

### Trajectory analysis

All MD systems become stable after 10 ns simulation, so the final analyses were performed using the 10–200 ns trajectories. For each system, trajectories from five independent simulations were combined together. Saved snapshots were analyzed using *cpptraj* module in AmberTools 15. All figures and videos are produced using Pymol^62^, Chimera^63^, and Microsoft Excel.

### Binding pocket analysis

Binding pocket analysis was performed using *AlphaSpace*, a computational tool for fragment-centric topographical mapping of intermolecular interfaces^35^. *AlphaSpace* utilizes a geometric model based on Voronoi tessellation to identify and represent all concave interaction space across the protein surface as a set of alpha-atom/alpha-space pairs, which are then clustered into discrete fragment-centric pockets. Pockets are selected for analysis based on direct contact with any atom from the peptide ligand. The occupation status of each individual alpha-space within each pocket is evaluated based on the distance between its associated alpha-atom and the nearest atom from the peptide ligand, using a 1.6 Å cutoff. Each occupied alpha-space is associated with the residue from the peptide containing the ligand atom nearest to that alpha-atom. The total pocket occupation by residue is calculated by taking the sum of all occupied alpha-space volumes associated with that residue.

## Additional Information

**How to cite this article**: Hou, X. *et al*. Resveratrol Serves as a Protein-Substrate Interaction Stabilizer in Human SIRT1 Activation. *Sci. Rep.* **6**, 38186; doi: 10.1038/srep38186 (2016).

**Publisher's note:** Springer Nature remains neutral with regard to jurisdictional claims in published maps and institutional affiliations.

## Supplementary Material

Supplementary Information

Supplementary Video 1

Supplementary Video 2

Supplementary Video 3

Supplementary Video 4

Supplementary Video 5

Supplementary Video 6

## Figures and Tables

**Table 1:** Comparison of substrate sequences, SIRT1 activation effects, and substrate binding affinities from MD simulations with three distinct p53-derived substrates.

Substrate	Sequence	SIRT1 activation by resveratrol^27^,^28^,^29^,^31^,^34^	Substrate binding from MD simulations	
			−Resveratrol	+Resveratrol
p53AMC	{RHKK(ac)AMC}	Yes	Loose	Tight
p53W	{RHKK(ac)W}	Yes	Loose	Tight
p53	{RHKK(ac)LMFKT}	No	Tight	Unchanged

## References

[b1] Imai S. & Guarente L. NAD(+) and sirtuins in aging and disease. Trends in Cell Biol. 24, 464–471 (2014). 24786309 10.1016/j.tcb.2014.04.002 PMC4112140

[b2] Sinclair D. A. & Guarente L. Small-Molecule Allosteric Activators of Sirtuins. Annu. Rev. Pharmacol. 54, 363–380 (2014). 10.1146/annurev-pharmtox-010611-134657 PMC4018738 24160699

[b3] Guarente L. Sirtuins and ageing-new findings. EMBO Rep. 14, 750–750 (2013). 23928812 10.1038/embor.2013.121 PMC3790060

[b4] Hubbard B. P. & Sinclair D. A. Small molecule SIRT1 activators for the treatment of aging and age-related diseases. Trends Pharmacol. Sci. 35, 146–154 (2014). 24439680 10.1016/j.tips.2013.12.004 PMC3970218

[b5] Guarente L. Calorie restriction and sirtuins revisited. Genes Dev. 27, 2072–2085 (2013). 24115767 10.1101/gad.227439.113 PMC3850092

[b6] Guarente L. & Picard F. Calorie restriction - the SIR2 connection. Cell 120, 473–482 (2005). 15734680 10.1016/j.cell.2005.01.029

[b7] Baur J. A., Ungvari Z., Minor R. K., Le Couteur D. G. & de Cabo R. Are sirtuins viable targets for improving healthspan and lifespan? Nat. Rev. Drug Discovery 11, 443–461 (2012). 22653216 10.1038/nrd3738 PMC4684642

[b8] Chen D., Steele A. D., Lindquist S. & Guarente L. Increase in activity during calorie restriction requires Sirt1. Science 310, 1641–1641 (2005). 16339438 10.1126/science.1118357

[b9] Banks A. S. . SirT1 Gain of Function Increases Energy Efficiency and Prevents Diabetes in Mice. Cell Metab. 8, 333–341 (2008). 18840364 10.1016/j.cmet.2008.08.014 PMC3222897

[b10] Bordone L. . SIRT1 transgenic mice show phenotypes resembling calorie restriction. Aging Cell 6, 759–767 (2007). 17877786 10.1111/j.1474-9726.2007.00335.x

[b11] Satoh A. . Sirt1 Extends Life Span and Delays Aging in Mice through the Regulation of Nk2 Homeobox 1 in the DMH and LH. Cell Metab. 18, 416–430 (2013). 24011076 10.1016/j.cmet.2013.07.013 PMC3794712

[b12] Herranz D. . Sirt1 improves healthy ageing and protects from metabolic syndrome-associated cancer. Nat. Commun. 1, 3 (2010). 20975665 10.1038/ncomms1001 PMC3641391

[b13] Satoh A. & Imai S. I. Systemic regulation of mammalian ageing and longevity by brain sirtuins. Nat. Commun. 5, 4211 (2014). 24967620 10.1038/ncomms5211 PMC4521907

[b14] Cohen H. Y. . Calorie restriction promotes mammalian cell survival by inducing the SIRT1 deacetylase. Science 305, 390–392 (2004). 15205477 10.1126/science.1099196

[b15] Chen D. . Tissue-specific regulation of SIRT1 by calorie restriction. Genes Dev. 22, 1753–1757 (2008). 18550784 10.1101/gad.1650608 PMC2492662

[b16] Rodgers J. T. . Nutrient control of glucose homeostasis through a complex of PGC-1 alpha and SIRT1. Nature 434, 113–118 (2005). 15744310 10.1038/nature03354

[b17] Schenk S. . Sirt1 enhances skeletal muscle insulin sensitivity in mice during caloric restriction. J. Clin. Invest. 121, 4281–4288 (2011). 21985785 10.1172/JCI58554 PMC3204844

[b18] Kume S. . Calorie restriction enhances cell adaptation to hypoxia through Sirt1-dependent mitochondrial autophagy in mouse aged kidney. J. Clin. Invest. 120, 1043–1055 (2010). 20335657 10.1172/JCI41376 PMC2846062

[b19] IBaur J. A. & Sinclair D. A. Therapeutic potential of resveratrol: the *in vivo* evidence. Nat. Rev. Drug Discovery 5, 493–506 (2006). 16732220 10.1038/nrd2060

[b20] Diaz-Gerevini G. T. . Beneficial action of resveratrol: How and why? Nutrition 32, 174–178 (2016). 26706021 10.1016/j.nut.2015.08.017

[b21] Price N. L. . SIRT1 Is Required for AMPK Activation and the Beneficial Effects of Resveratrol on Mitochondrial Function. Cell Metab. 15, 675–690 (2012). 22560220 10.1016/j.cmet.2012.04.003 PMC3545644

[b22] Tennen R. I., Michishita-Kioi E. & Chua K. F. Finding a Target for Resveratrol. Cell 148, 387–389 (2012). 22304906 10.1016/j.cell.2012.01.032

[b23] Chung J. H., Manganiello V. & Dyck J. R. B. Resveratrol as a calorie restriction mimetic: therapeutic implications. Trends in Cell Biol. 22, 546–554 (2012). 22885100 10.1016/j.tcb.2012.07.004 PMC3462230

[b24] Howitz K. T. . Small molecule activators of sirtuins extend Saccharomyces cerevisiae lifespan. Nature 425, 191–196 (2003). 12939617 10.1038/nature01960

[b25] IWood J. G. . Sirtuin activators mimic caloric restriction and delay ageing in metazoans. Nature 430, 686–689 (2004). 15254550 10.1038/nature02789

[b26] Baur J. A. . Resveratrol improves health and survival of mice on a high-calorie diet. Nature 444, 337–342 (2006). 17086191 10.1038/nature05354 PMC4990206

[b27] Kaeberlein M. . Substrate-specific activation of sirtuins by resveratrol. J. Biol. Chem. 280, 17038–17045 (2005). 15684413 10.1074/jbc.M500655200

[b28] Borra M. T., Smith B. C. & Denu J. M. Mechanism of human SIRT1 activation by resveratrol. J. Biol. Chem. 280, 17187–17195 (2005). 15749705 10.1074/jbc.M501250200

[b29] Dai H. . SIRT1 activation by small molecules: kinetic and biophysical evidence for direct interaction of enzyme and activator. J. Biol. Chem. 285, 32695–32703 (2010). 20702418 10.1074/jbc.M110.133892 PMC2963390

[b30] Pacholec M. . SRT1720, SRT2183, SRT1460, and Resveratrol Are Not Direct Activators of SIRT1. J. Biol. Chem. 285, 8340–8351 (2010). 20061378 10.1074/jbc.M109.088682 PMC2832984

[b31] Cao D. F. . Structural basis for allosteric, substrate-dependent stimulation of SIRT1 activity by resveratrol. Genes Dev. 29, 1316–1325 (2015). 26109052 10.1101/gad.265462.115 PMC4495401

[b32] Dai H. . Crystallographic structure of a small molecule SIRT1 activator-enzyme complex. Nat. Commun. 6, 7645 (2015). 26134520 10.1038/ncomms8645 PMC4506539

[b33] Lakshminarasimhan M., Rauh D., Schutkowski M. & Steegborn C. Sirt1 activation by resveratrol is substrate sequence-selective. Aging 5, 151–154 (2013). 23524286 10.18632/aging.100542 PMC3629287

[b34] Hubbard B. P. . Evidence for a Common Mechanism of SIRT1 Regulation by Allosteric Activators. Science 339, 1216–1219 (2013). 23471411 10.1126/science.1231097 PMC3799917

[b35] Rooklin D., Wang C., Katigbak J., Arora P. S. & Zhang Y. AlphaSpace: Fragment-Centric Topographical Mapping To Target Protein-Protein Interaction Interfaces. J. Chem. Inf. Model. 55, 1585–1599 (2015). 26225450 10.1021/acs.jcim.5b00103 PMC4550072

[b36] Milne J. C. . Small molecule activators of SIRT1 as therapeutics for the treatment of type 2 diabetes. Nature 450, 712–716 (2007). 18046409 10.1038/nature06261 PMC2753457

[b37] Cosgrove M. S. . The structural basis of sirtuin substrate affinity. Biochemistry 45, 7511–7521 (2006). 16768447 10.1021/bi0526332

[b38] Avalos J. L. . Structure of a Sir2 enzyme bound to an acetylated p53 peptide. Mol. Cell 10, 523–535 (2002). 12408821 10.1016/s1097-2765(02)00628-7

[b39] Gut P. & Verdin E. Rejuvenating SIRT1 Activators. Cell Metab. 17, 635–637 (2013). 23663735 10.1016/j.cmet.2013.04.016

[b40] Liu B. H. . Resveratrol Rescues SIRT1-Dependent Adult Stem Cell Decline and Alleviates Progeroid Features in Laminopathy-Based Progeria. Cell Metab. 16, 738–750 (2012). 23217256 10.1016/j.cmet.2012.11.007

[b41] Kim E. J., Kho J. H., Kang M. R. & Um S. J. Active regulator of SIRT1 cooperates with SIRT1 and facilitates suppression of p53 activity. Mol. Cell 28, 513–513 (2007). 10.1016/j.molcel.2007.08.030 17964266

[b42] Hsu H. C. . Structural basis for allosteric stimulation of Sir2 activity by Sir4 binding. Genes Dev. 27, 64–73 (2013). 23307867 10.1101/gad.208140.112 PMC3553284

[b43] Aeluri M. . Small Molecule Modulators of Protein-Protein Interactions: Selected Case Studies. Chem. Rev. 114, 4640–4694 (2014). 24673632 10.1021/cr4004049

[b44] Milroy L. G., Grossmann T. N., Hennig S., Brunsveld L. & Ottmann C. Modulators of Protein-Protein Interactions. Chem. Rev. 114, 4695–4748 (2014). 24735440 10.1021/cr400698c

[b45] Giordanetto F., Schafer A. & Ottmann C. Stabilization of protein-protein interactions by small molecules. Drug Discovery Today 19, 1812–1821 (2014). 25173701 10.1016/j.drudis.2014.08.005

[b46] Choi J. W., Chen J., Schreiber S. L. & Clardy J. Structure of the FKBP12-rapamycin complex interacting with the binding domain of human FRAP. Science 273, 239–242 (1996). 8662507 10.1126/science.273.5272.239

[b47] Liang J., Choi J. & Clardy J. Refined structure of the FKBP12-rapamycin-FRB ternary complex at 2.2 angstrom resolution. Acta Crystallogr. 55, 736–744 (1999). 10.1107/s0907444998014747 10089303

[b48] Yang H. J. . mTOR kinase structure, mechanism and regulation. Nature 497, 217–223 (2013). 23636326 10.1038/nature12122 PMC4512754

[b49] Aylett C. H. S. . Structural Biology Architecture of human mTOR complex 1. Science 351, 48–52 (2016). 26678875 10.1126/science.aaa3870

[b50] Dolinsky T. J., Nielsen J. E., McCammon J. A. & Baker N. A. PDB2PQR: an automated pipeline for the setup of Poisson-Boltzmann electrostatics calculations. Nucleic Acids Res. 32, W665–W667 (2004). 15215472 10.1093/nar/gkh381 PMC441519

[b51] Shi Y. W., Zhou Y. Z., Wang S. L. & Zhang Y. K. Sirtuin Deacetylation Mechanism and Catalytic Role of the Dynamic Cofactor Binding Loop. J. Phys. Chem. Lett. 4, 491–495 (2013). 23585919 10.1021/jz302015s PMC3621114

[b52] Case D. A. . AMBER 14, *University of California, San Francisco*, http://ambermd.org/ (2014).

[b53] Maier J. A. . ff14SB: Improving the Accuracy of Protein Side Chain and Backbone Parameters from ff99SB. J. Chem. Theory Comput. 11, 3696–3713 (2015). 26574453 10.1021/acs.jctc.5b00255 PMC4821407

[b54] Peters M. B. . Structural Survey of Zinc-Containing Proteins and Development of the Zinc AMBER Force Field (ZAFF). J. Chem. Theory Comput. 6, 2935–2947 (2010). 20856692 10.1021/ct1002626 PMC2941202

[b55] Papamokos G. V. . Structural Role of RKS Motifs in Chromatin Interactions: A Molecular Dynamics Study of HP1 Bound to a Variably Modified Histone Tail. Biophys. J. 102, 1926–1933 (2012). 22768949 10.1016/j.bpj.2012.03.030 PMC3328719

[b56] Ryde U. On the Role of Glu-68 in Alcohol-Dehydrogenase. Protein Sci. 4, 1124–1132 (1995). 7549877 10.1002/pro.5560040611 PMC2143135

[b57] Frisch M. J. . Gaussian 09. (Gaussian, Inc., Wallingford, CT, USA, 2009).

[b58] Cieplak P., Cornell W. D., Bayly C. & Kollman P. A. Application of the Multimolecule and Multiconformational Resp Methodology to Biopolymers - Charge Derivation for DNA, Rna, and Proteins. J. Comput. Chem. 16, 1357–1377 (1995).

[b59] Bayly C. I., Cieplak P., Cornell W. D. & Kollman P. A. A Well-Behaved Electrostatic Potential Based Method Using Charge Restraints for Deriving Atomic Charges - the Resp Model. J Phys. Chem. 97, 10269–10280 (1993).

[b60] Salomon-Ferrer R., Gotz A. W., Poole D., Le Grand S. & Walker R. C. Routine Microsecond Molecular Dynamics Simulations with AMBER on GPUs. 2. Explicit Solvent Particle Mesh Ewald. J. Chem. Theory Comput. 9, 3878–3888 (2013). 26592383 10.1021/ct400314y

[b61] Gotz A. W. . Routine Microsecond Molecular Dynamics Simulations with AMBER on GPUs. 1. Generalized Born. J. Chem. Theory Comput. 8, 1542–1555 (2012). 22582031 10.1021/ct200909j PMC3348677

[b62] Schrödinger L. The PyMOL Molecular Graphics System, Version 1.7. 4 Schrödinger, LLC.

[b63] Pettersen E. F. . UCSF Chimera--a visualization system for exploratory research and analysis. J. Comput. Chem. 25, 1605–1612 (2004). 15264254 10.1002/jcc.20084

