# Supplementary material for: Resveratrol serves as a protein-substrate interaction stabilizer in human SIRT1 activation
Source: Sci Rep. 2016 Nov 30;6:38186. doi: 10.1038/srep38186 (PMC5128864; doi:10.1038/srep38186)
Supplement: Supplementary Information [file srep38186-s1.pdf]

Supplementary Information

Resveratrol Serves as a Protein-Substrate Interaction Stabilizer in Human SIRT1 Activation

Xuben Hou[†, ‡], David Rooklin[‡], Hao Fang[†], Yingkai Zhang[*, ‡, §]

[†]Department of Medicinal Chemistry and Key Laboratory of Chemical Biology of Natural Products (MOE), School of Pharmacy, Shandong University, Jinan, Shandong 250012, China

[‡]Department of Chemistry, New York University, New York, New York 10003 United States

[§]NYU-ECNU Center for Computational Chemistry at NYU Shanghai, Shanghai 200062, China

Correspondence and requests for materials should be addressed to Y.Z. (email: yingkai.zhang@nyu.edu).

The following supporting information includes Tables S1, Figures S1-S11, details for minimizations and equilibrations in MD simulations, details for MM/GBSA calculations and legends for Video S1-S6.

Table S1. Summary of MD simulations.

#	Duration (µs)	Protein	Substrate	Activator
**1**	0.2	SIRT1	p53AMC	-
**2**	0.2	SIRT1	p53AMC	-
**3**	0.2	SIRT1	p53AMC	-
**4**	0.2	SIRT1	p53AMC	-
**5**	0.2	SIRT1	p53AMC	-
**6**	0.2	SIRT1	p53AMC	RSV 1+2+3
**7**	0.2	SIRT1	p53AMC	RSV 1+2+3
**8**	0.2	SIRT1	p53AMC	RSV 1+2+3
**9**	0.2	SIRT1	p53AMC	RSV 1+2+3
**10**	0.2	SIRT1	p53AMC	RSV 1+2+3
**11**	0.2	SIRT1	p53W	-
**12**	0.2	SIRT1	p53W	-
**13**	0.2	SIRT1	p53W	-
**14**	0.2	SIRT1	p53W	-
**15**	0.2	SIRT1	p53W	-
**16**	0.2	SIRT1	p53W	RSV 1+2+3
**17**	0.2	SIRT1	p53W	RSV 1+2+3
**18**	0.2	SIRT1	p53W	RSV 1+2+3
**19**	0.2	SIRT1	p53W	RSV 1+2+3
**20**	0.2	SIRT1	p53W	RSV 1+2+3
**21**	0.2	SIRT1	p53	-
**22**	0.2	SIRT1	p53	-
**23**	0.2	SIRT1	p53	-
**24**	0.2	SIRT1	p53	-
**25**	0.2	SIRT1	p53	-
**26**	0.2	SIRT1	p53	RSV 1+2+3
**27**	0.2	SIRT1	p53	RSV 1+2+3
**28**	0.2	SIRT1	p53	RSV 1+2+3
**29**	0.2	SIRT1	p53	RSV 1+2+3
**30**	0.2	SIRT1	p53	RSV 1+2+3
**31**	0.2	SIRT1	-	-
**32**	0.2	SIRT1	-	-
**33**	0.2	SIRT1	-	-
**34**	0.2	SIRT1	-	-
**35**	0.2	SIRT1	-	-

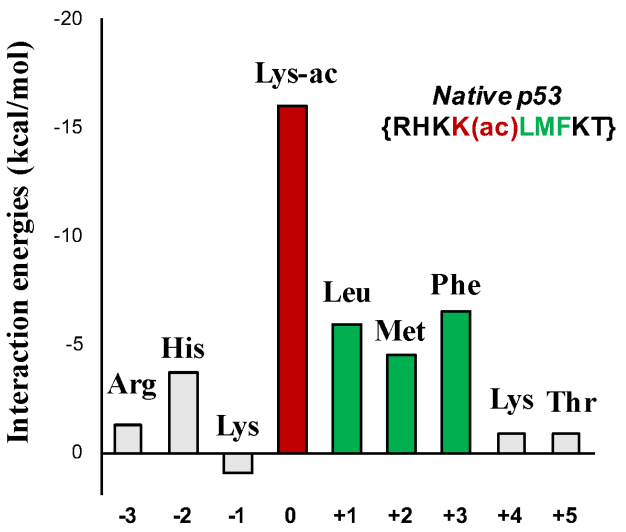

Figure S1. Sequence of native p53 substrate and the average interaction energies on SIRT1, by residue. The data bar of Lys-ac is highlighted in red and that of Leu (+1), Met (+2) and Phe (+3) are highlighted in green. Calculations were performed using MM/GBSA method (see page S12).

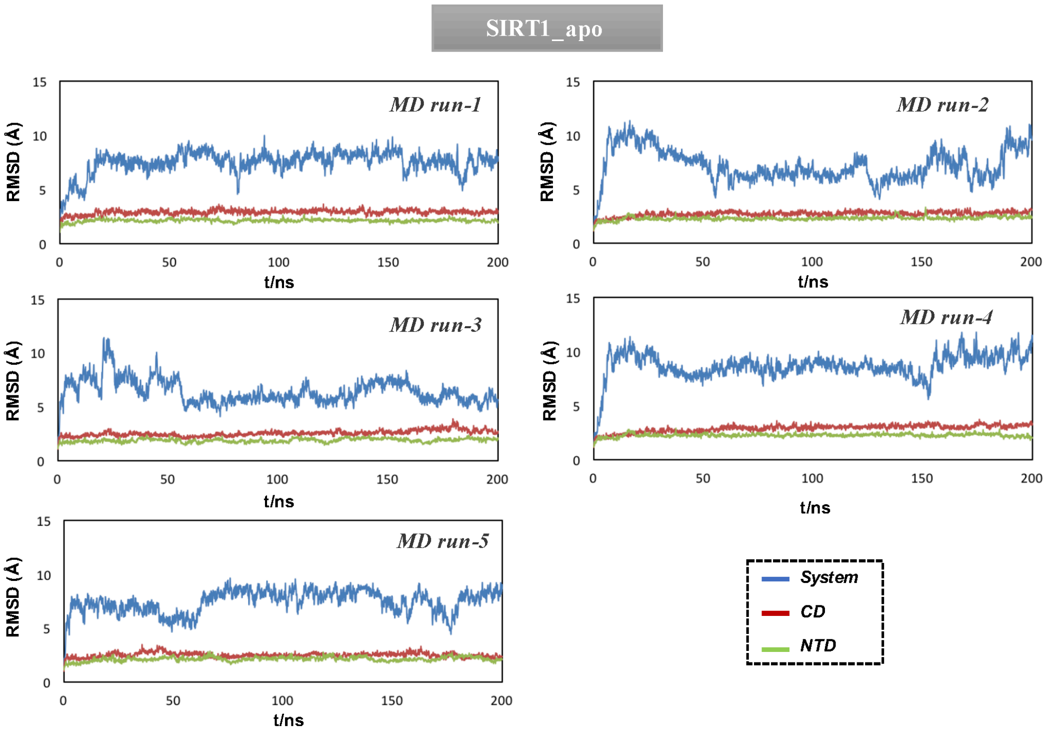

Figure S2. The RMSD values of total system (blue), CD (red) and NTD (green) during 5 independent MD runs of SIRT1_apo system.

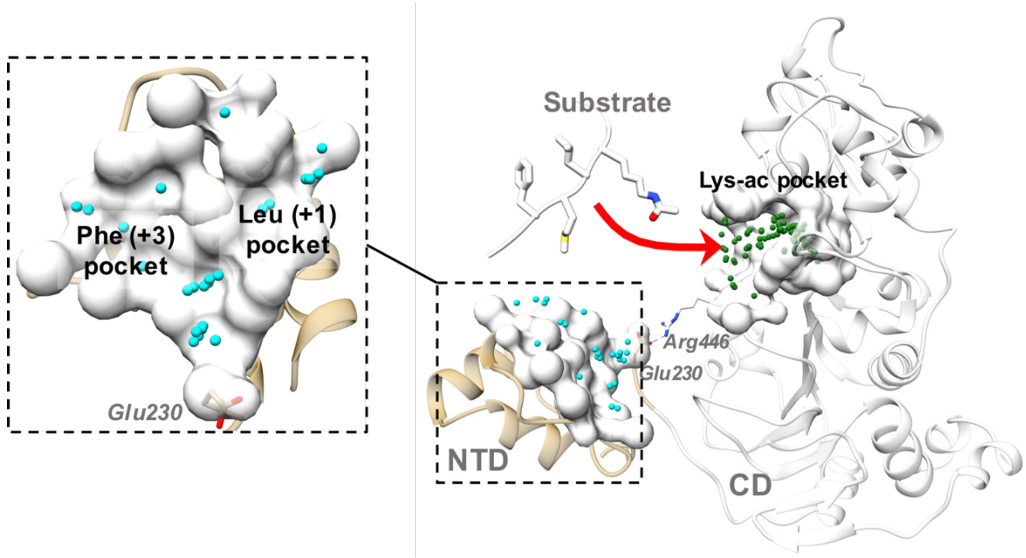

Figure S3. Mapping of Lys-ac pocket in CD (green sphere) and Leu/Phe pockets in NTD (cyan sphere) from the unbound state of SIRT1. SIRT1 is shown in cartoon with NTD colored in tan.

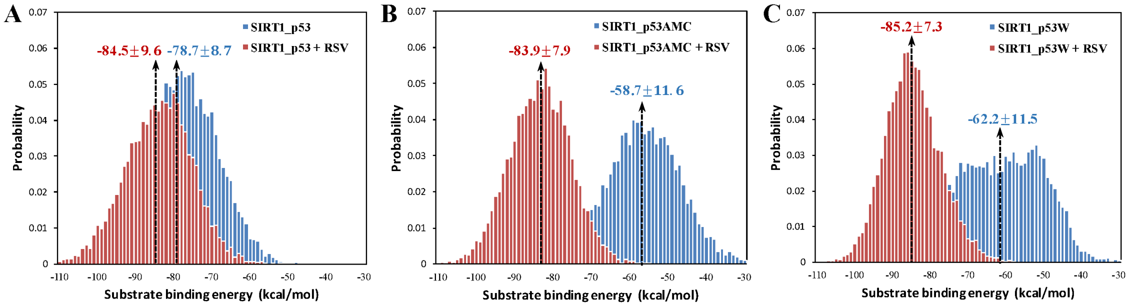

Figure S4. Calculated interactions energies between SIRT1 and substrate p53 (A), p53AMC(B) and p53W(C) with or without resveratrol bound using MM/GBSA method (see page S12).

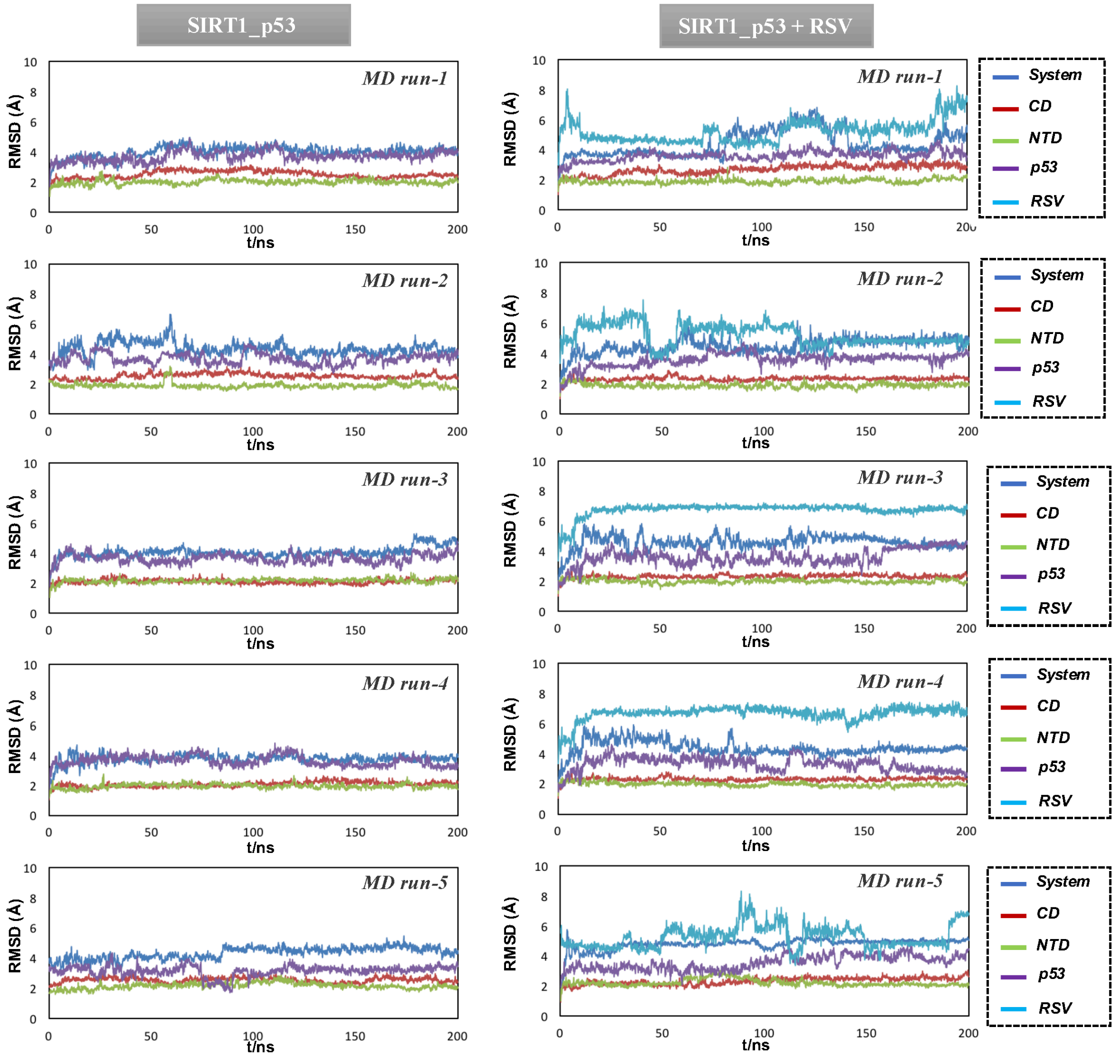

Figure S5. The RMSD values of total system (blue), CD (red), NTD (green), substrate (purple) and resveratrol (cyan) during 5 independent MD runs of SIRT1_p53 system with or without resveratrol bound.

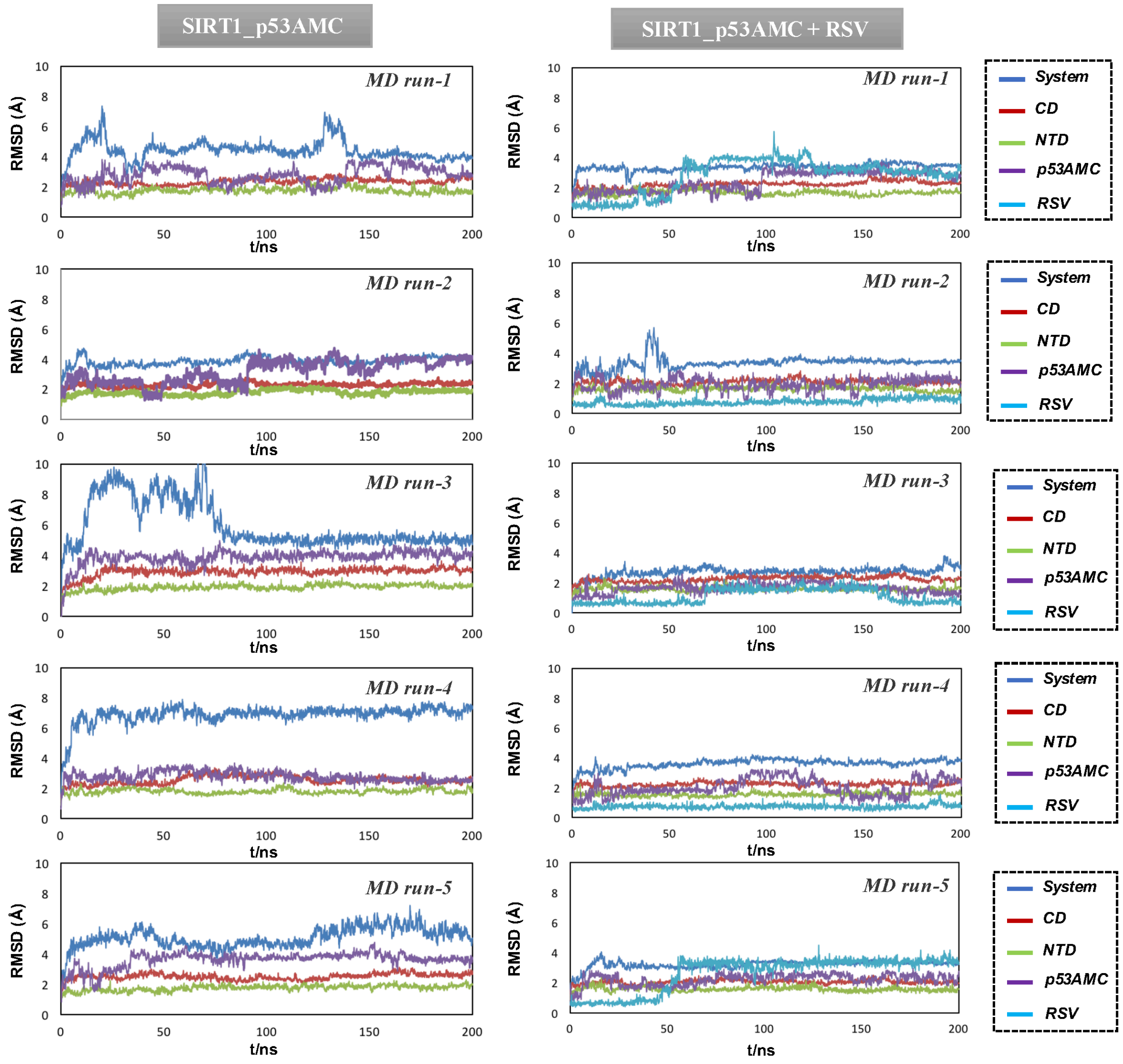

Figure S6. The RMSD values of total system (blue), CD (red), NTD (green), substrate (purple) and resveratrol (cyan) during 5 independent MD runs of SIRT1_p53AMC system with or without resveratrol bound.

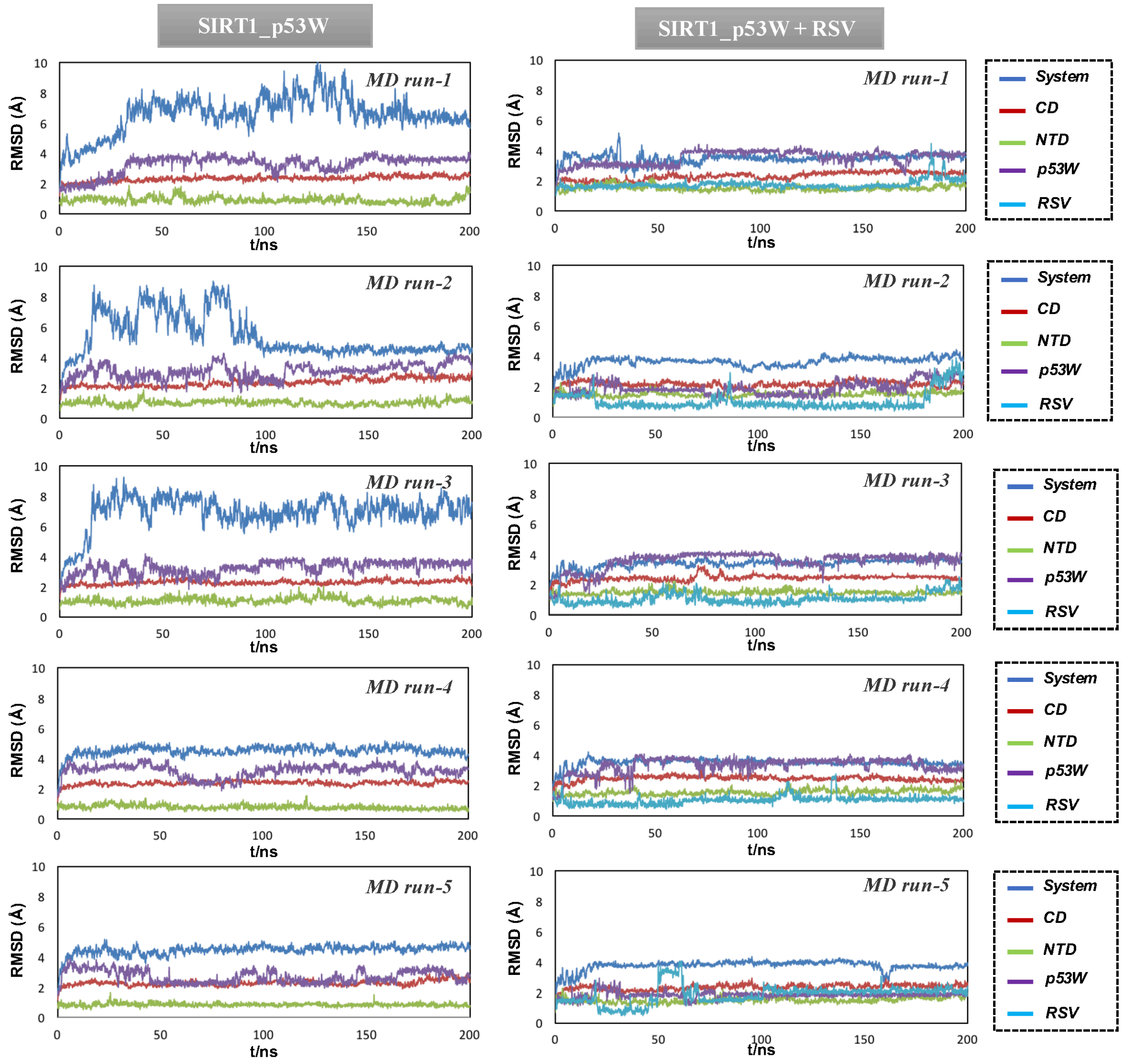

Figure S7. The RMSD values of total system (blue), CD (red), NTD (green), substrate (purple) and resveratrol (cyan) during 5 independent MD runs of SIRT1_p53W system with or without resveratrol bound.

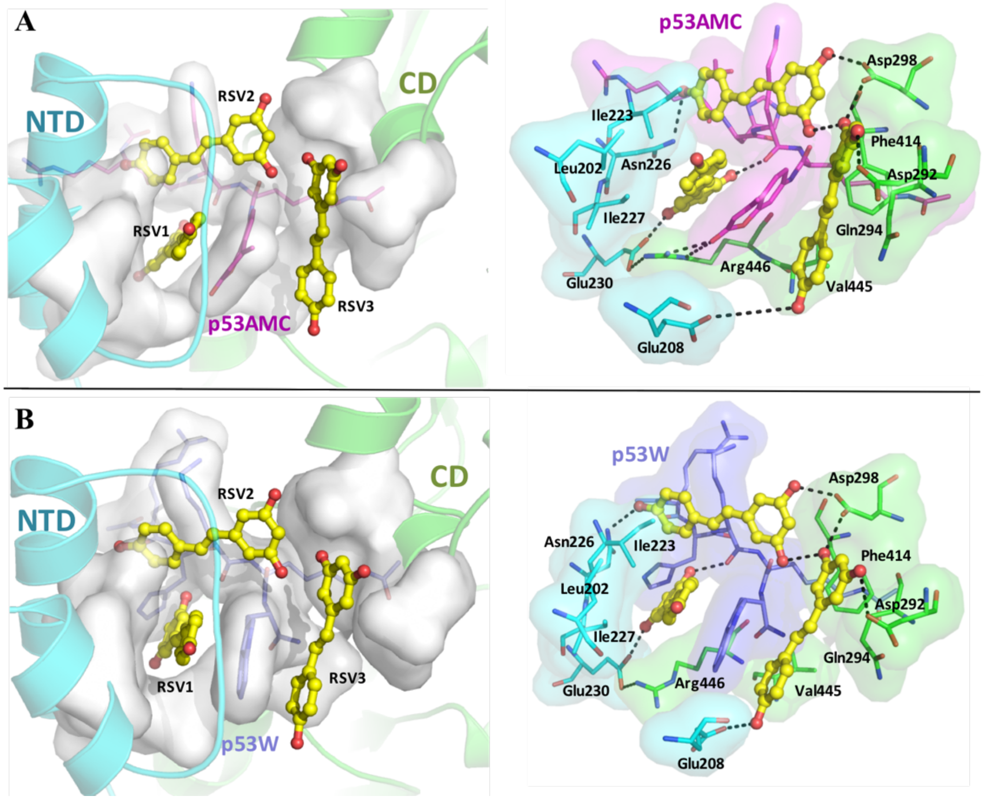

Figure S8. (A) The binding of three resveratrol in SIRT1-p53AMC complex. (B) The binding of three resveratrol in SIRT1-p53W complex. Hydrogen bonds are illustrated in black dotted line. Residue Glu230 in NTD can form salt-bridge interaction with Arg446 in CD.

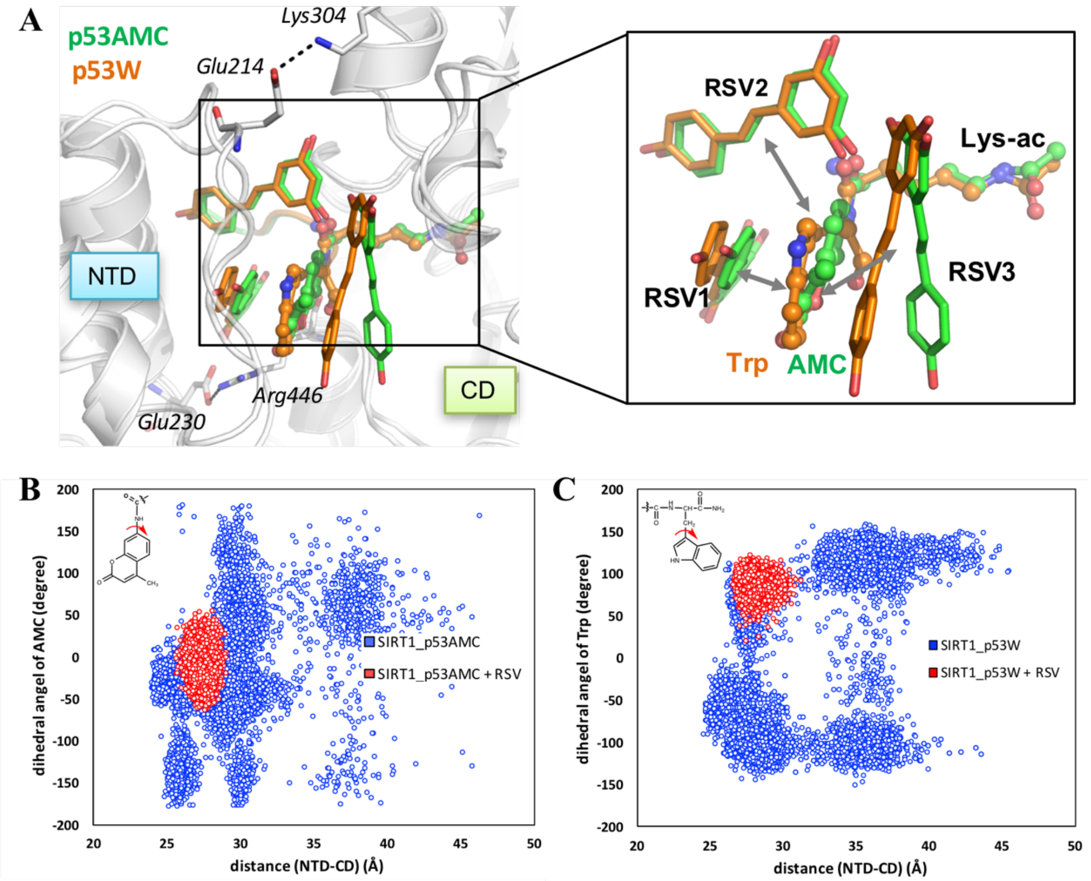

Figure S9. (A) Comparison of binding conformations of p53AMC and p53W with resveratrol bound. (B) Correlations of AMC dihedral angle with NTD-CD distance. (C) Correlations of Trp indole ring dihedral angle with NTD-CD distance.

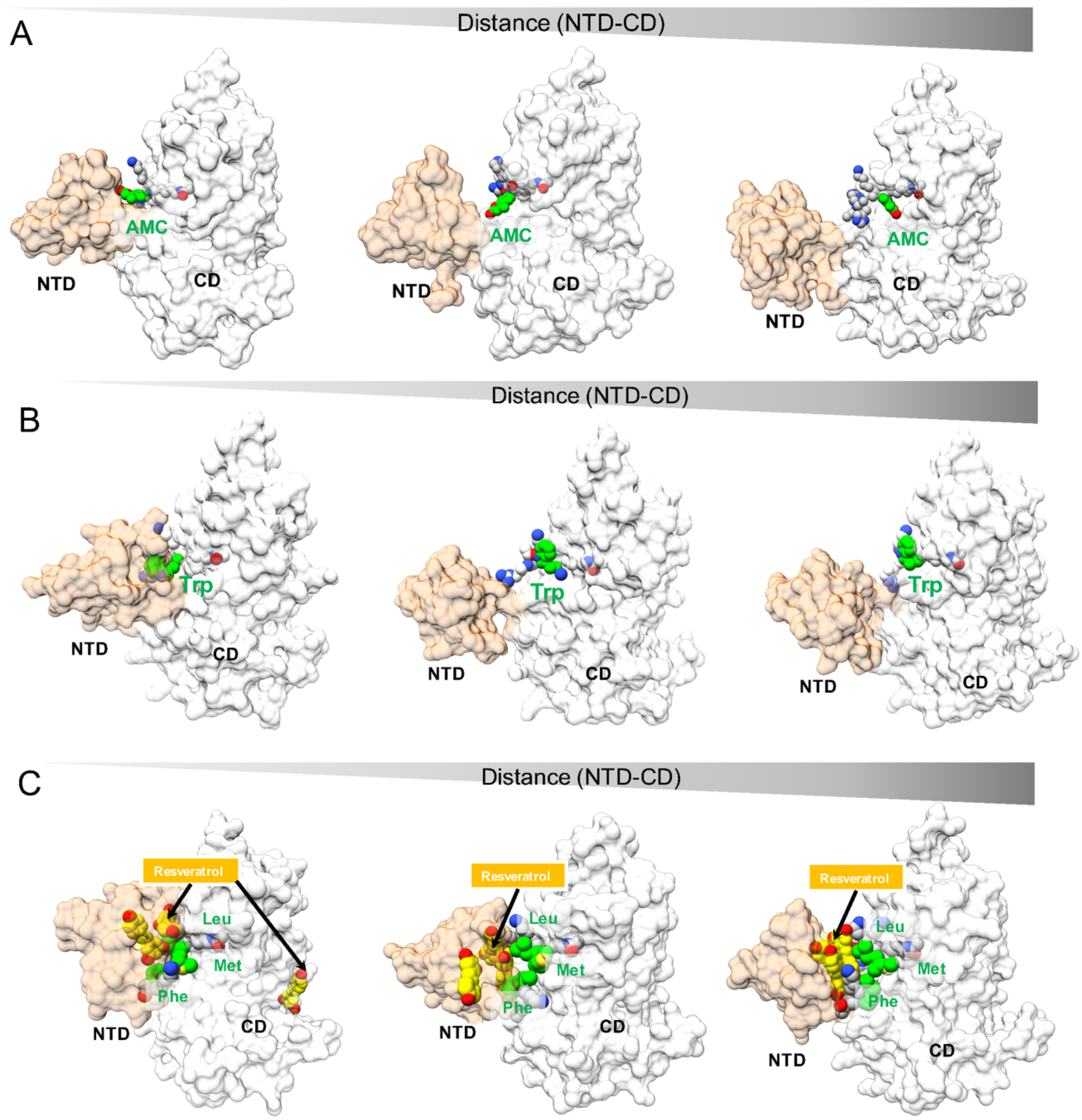

Figure S10. Representative conformations of SIRT1-p53AMC (A), SIRT1-p53W (B) and SIRT1-p53 with resveratrol bound (C) from clustering analysis based on distance between NTD and CD. Substrates are presented in grey sphere with specific motifs (AMC in p53AMC, Trp in p53W and Leu, Met and Phe in p53) colored in green. Resveratrol are presented in yellow sphere.

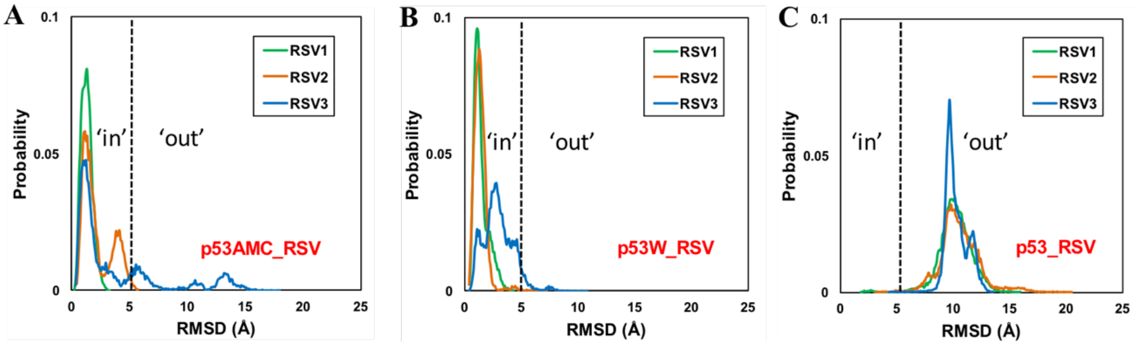

Figure S11. Distributions of RMSD values of three resveratrol (from crystal 5BTR[1]) during MD simulations using p53AMC(A), p53W(B) and p53(C). RMSD values were calculated with MD snapshots superposed using the backbone atoms of CD (catalytic domain).

Additional computational methods.

Minimizations and equilibrations.

First, 2500 steps of steepest descent minimization followed by 2500 cycles of conjugate gradient minimization were conducted on the hydrogen atoms, water molecules and counterions with a restraint force constant of 500 kcal/(mol·Å^2) on the heavy atoms of protein. Then, 2500 steps of steepest descent minimization followed by 2500 cycles of conjugate gradient minimization were carried out on the water molecules and counterions with a restraint force constant of 50 kcal/(mol·Å^2) on the heavy atoms of protein.

The minimized structure was then subjected to five rounds of equilibration. First, each system was equilibrated at constant temperature of 10K for 25ps simulation with the solute molecules fixed with a restraint force constant of 50 kcal/(mol·Å^2) at constant pressure. Then the system was heated from 10 K to 300 K over 50 ps with the solute molecules fixed with a restraint force constant of 50 kcal/(mol·Å^2) at constant volume. Then the system was equilibrated at constant temperature of 300K for 25ps with a restraint force constant of 50 kcal/(mol·Å^2) at constant volume. In the last round of equilibration, the restraint force constant on the solute was reduced through three steps: at 50 kcal/(mol·Å^2) for 50 ps, at 10 kcal/(mol·Å^2) for 50 ps, then at 1 kcal/(mol·Å^2) for 50 ps with constant pressure at 300K.

Calculations of substrates interaction energies.

The widely used molecular mechanics/generalized born solvent accessibility (MM/GBSA) methodology was employed to calculate the relative binding energies of substrates in different simulation systems. MM-GBSA calculations were performed by *MM-PBSA.py* module of Amber14. For each system, SIRT1-substrate interaction energies were calculated using 9500 frames taken from 5 independent MD runs (10-200 ns) at intervals of 100 ps.

In the MM/GBSA methodology, the binding energies (ΔG_{bind}) are calculated as the sum of molecular mechanical and solvation energies as described by following equations:

$$\Delta G_{bind} = \Delta H + T\Delta S \approx \Delta E_{MM} + \Delta G_{sol} - T\Delta S \qquad (1)$$

where ΔE_{MM} is the gas phase molecular mechanical energy; ΔG_{sol} is the desolvation free energy; $-T\Delta S$ represent the conformational entropy upon association of substrata at

temperature T. Due to the expensive computational cost and low prediction accuracy2-5, entropies were not considered in current study.

$$\Delta E_{MM} = \Delta E_{internal} + \Delta E_{electrostatic} + \Delta E_{vdm} \qquad (2)$$

According eq 2., the ΔE_{MM} contains the internal energy term ($\Delta E_{internal}$, the summation of bond, angle, and dihedral energies), the electrostatic energy term ($\Delta E_{electrostatic}$), and the van der Waals energy term (ΔE_{vdm}). The solvation energy is calculated according to eq 3.

$$\Delta G_{sol} = \Delta G_{GB} + \Delta G_{SA} \qquad (3)$$

where ΔG_{GB} is the electrostatic solvation energy and ΔG_{SA} is the nonpolar solvation component. The electrostatic solvation energy (ΔG_{GB}) was calculated by the generalized born (GB) model developed by Onufriev et al [6-7]. and the nonpolar component is determined by solvent accessible surface area (SASA) uses a fast LCPO algorithm8. The physiological salt concentration of 0.15 M was employed for all MM/GBSA calculations.

Captions for the six movie files.

Video S1. Movies of representative MD simulation of SIRT1_apo system. SIRT1 is presented in ribbon with NTD colored in cyan and CD colored in green. Residues that form salt-bridge interactions between NTD and CD are shown in sticks and the distances are shown as dotted line. The NAD^+ is presented as white stick.

Video S2. Movies of representative MD simulation of SIRT1_p53 system. SIRT1 is presented in ribbon with NTD colored in cyan and CD colored in green. Substrate p53 is shown as grey ribbon with key residues presented in sticks (Lys-ac is colored in grey; Leu, Met and Phe are colored in red). Residues that form salt-bridge interactions between NTD and CD are shown in sticks and the distances are shown as dotted line. The NAD^+ is presented as white stick.

Video S3. Movies of representative MD simulation of SIRT1_p53AMC system. SIRT1 is presented in ribbon with NTD colored in cyan and CD colored in green. Substrate p53AMC is shown as grey ribbon with key residues presented in sticks (Lys-ac is colored in grey; AMC is colored in red). Residues that form salt-bridge interactions between NTD and CD are shown in sticks and the distances are shown as dotted line. The NAD^+ is presented as white stick.

Video S4. Movies of representative MD simulation of SIRT1_p53AMC + RSV system. SIRT1 is presented in ribbon with NTD colored in cyan and CD colored in green. Substrate p53AMC is shown as grey ribbon with key residues presented in sticks (Lys-ac is colored in grey; AMC is colored in red). Three resveratrol molecules are shown as yellow sticks. Residues that form salt-bridge interactions between NTD and CD are shown in sticks and the distances are shown as dotted line. The NAD^+ is presented as white stick.

Video S5. Movies of representative MD simulation of SIRT1_p53W system. SIRT1 is presented in ribbon with NTD colored in cyan and CD colored in green. Substrate p53W is shown as grey ribbon with key residues presented in sticks (Lys-ac is colored in grey; Trp is colored in red). Residues that form salt-bridge interactions between NTD and CD are shown in sticks and the distances are shown as dotted line. The NAD^+ is presented as white stick.

Video S6. Movies of representative MD simulation of SIRT1_p53W + RSV system. SIRT1 is presented in ribbon with NTD colored in cyan and CD colored in green. Substrate p53W is shown as grey ribbon with key residues presented in sticks (Lys-ac is colored in grey; Trp is colored in red). Three resveratrol molecules are shown as yellow sticks. Residues that form salt-bridge interactions between NTD and CD are shown in sticks and the distances are shown as dotted line. The NAD^+ is presented as white stick.

References

(1) Cao, D. F.; Wang, M. Z.; Qiu, X. Y.; Liu, D. X.; Jiang, H. L.; Yang, N.; Xu, R. M., *Gene Dev*. **2015,** *29*, 1316.

(2) Hou, T. J.; Wang, J. M.; Li, Y. Y.; Wang, W., *J. Comput. Chem*. **2011,** *32*, 866.

(3) Genheden, S.; Kuhn, O.; Mikulskis, P.; Hoffmann, D.; Ryde, U., *J. Chem. Inf. Model*. **2012,** *52*, 2079.

(4) Hou, T. J.; Wang, J. M.; Li, Y. Y.; Wang, W., *J. Chem. Inf. Model*. **2011,** *51*, 69.

(5) Li, L.; Li, Y. Y.; Zhang, L. L.; Hou, T. J., *J. Chem. Inf. Model*. **2012,** *52*, 2715.

(6) Onufriev, A.; Bashford, D.; Case, D. A., *Proteins* **2004,** *55*, 383.

(7) Feig, M.; Onufriev, A.; Lee, M. S.; Im, W.; Case, D. A.; Brooks, C. L., *J. Comput. Chem*. **2004,** *25*, 265.

(8) Weiser, J.; Shenkin, P. S.; Still, W. C., *J. Comput. Chem*. **1999,** *20*, 217.
